# Health service quality in 2929 facilities in six low-income and middle-income countries: a positive deviance analysis

**DOI:** 10.1016/S2214-109X(23)00163-8

**Published:** 2023-05-16

**Authors:** Todd P Lewis, Margaret McConnell, Amit Aryal, Grace Irimu, Suresh Mehata, Mwifadhi Mrisho, Margaret E Kruk

**Affiliations:** a Department of Global Health and Population, Harvard TH Chan School of Public Health, Boston, MA, USA; b Swiss TPH, University of Basel, Basel, Switzerland; c Department of Paediatrics and Child Health, University of Nairobi, Nairobi, Kenya; d Policy, Planning and Public Health Division, Ministry of Health, Biratnagar, Nepal; e Department of Health Systems, Impact Evaluation, and Policy, Ifakara Health Institute, Dar es Salaam, Tanzania

## Abstract

**Background:**

Primary care is of insufficient quality in many low-income and middle-income countries. Some health facilities perform better than others despite operating in similar contexts, although the factors that characterise best performance are not well known. Existing best-performance analyses are concentrated in high-income countries and focus on hospitals. We used the positive deviance approach to identify the factors that differentiate best from worst primary care performance among health facilities across six low-resource health systems.

**Methods:**

This positive deviance analysis used nationally representative samples of public and private health facilities from Service Provision Assessments of the Democratic Republic of the Congo, Haiti, Malawi, Nepal, Senegal, and Tanzania. Data were collected starting June 11, 2013, in Malawi and ending Feb 28, 2020, in Senegal. We assessed facility performance through completion of the Good Medical Practice Index (GMPI) of essential clinical actions (eg, taking a thorough history, conducting an adequate physical examination) according to clinical guidelines and measured with direct observations of care. We identified hospitals and clinics in the top decile of performance (defined as best performers) and conducted a quantitative, cross-national positive deviance analysis to compare them with facilities performing below the median (defined as worst performers) and identify facility-level factors that explain the gap between best and worst performance.

**Findings:**

We identified 132 best-performing and 664 worst-performing hospitals, and 355 best-performing and 1778 worst-performing clinics based on clinical performance across countries. The mean GMPI score was 0·81 (SD 0·07) for the best-performing hospitals and 0·44 (0·09) for the worst-performing hospitals. Among clinics, mean GMPI scores were 0·75 (0·07) for the best performers and 0·34 (0·10) for the worst performers. High-quality governance, management, and community engagement were associated with best performance compared with worst performance. Private facilities out-performed government-owned hospitals and clinics.

**Interpretation:**

Our findings suggest that best-performing health facilities are characterised by good management and leaders who can engage staff and community members. Governments should look to best performers to identify scalable practices and conditions for success that can improve primary care quality overall and decrease quality gaps between health facilities.

**Funding:**

Bill & Melinda Gates Foundation.

## Introduction

Primary care is an essential service delivery platform within the health system.^1^ It can reduce morbidity, increase patient longevity, and improve health equity.^2^ A functioning primary care service is crucial for detecting and treating infectious diseases and managing the growing burden of chronic illness facing low-income and middle-income countries (LMICs).^3^ The 2018 Declaration of Astana reaffirmed the essential role of primary care in building strong health systems and achieving the Sustainable Development Goals.^4^ The global pursuit of universal health coverage will also require access to affordable, high-quality primary care for all people.^5^

The quality of primary care services is not always adequate to optimise health.^6^ Poor quality health systems result in more than 8 million deaths from treatable conditions per year in LMICs, many of which can be treated in primary care.^6^ Studies show poor adherence to clinical guidelines among health-care workers in LMICs, who carry out on average just over half of recommended care actions during adult and child visits for primary care conditions.^7^ Low patient safety, limited detection and prevention functions, and poor user experience also undermine the effect of primary care on health outcomes.^6^ Primary care quality requires attention at multiple levels of the health system because people in LMICs commonly use hospitals as a sole source of essential health services.^8^

Data from direct observations of care in LMICs show technical quality varies within countries, with some facilities substantially outperforming others.^9^ This suggests higher quality care is obtainable in settings with similar resource constraints.^6^ Multiple factors influence variation in facility performance, including the underlying strength of the local health system in which they operate.^6^, ^9^, ^10^ However, knowledge gaps remain regarding the facility-level and contextual factors that drive variation in primary care quality in LMICs.^11^ More evidence is needed to identify actionable and modifiable facility-level characteristics to improve the quality of care delivery.

**Research in context**

**Evidence before this study**

We searched PubMed and Google Scholar for studies regarding performance drivers and key characteristics associated with excellent health facility performance in primary care. We used keywords such as “primary care”, “factor OR driver OR characteristic”, and “quality of care OR performance”, and performance characteristics such as “management”, “governance”, and “health worker”. We also searched for positive deviance and best performer analyses and conducted searches specifically focused on low-resource health systems. The search was limited to articles published in English between Jan 1, 2000, and Aug 1, 2021. The available literature was largely qualitative in nature and focused on secondary and tertiary health facilities in high-income settings. Few positive deviance analyses specifically examine primary care in low-income health systems. Findings from these studies suggest health facility management and basic leadership competencies are crucial for high-quality primary care. The available literature would be strengthened by quantitative analysis that includes facilities at multiple levels of the health system, especially in low-income and middle-income countries.

**Added value of this study**

In this study, we build on existing qualitative positive deviance work by quantitatively assessing the associations between performance indicators and best versus worst performance using large-scale, nationally representative health system surveys. We expand the primary care literature through application of positive deviance methods to direct observations of care separately among primary-level and secondary-level facilities. Our study demonstrates substantial performance gaps between facilities across countries despite operating in similar contexts. Findings show that strong management, community engagement, and maintenance of essential resources were associated with best performance and helped explain gaps between best and worst performance, especially among lower-level facilities. Among measured factors, only available supplies and equipment explained differential performance among hospitals. Private ownership was also associated with status as a best performer among both hospitals and clinics.

**Implications of all the available evidence**

Despite widespread variation in primary care performance, good medical practice is possible in resource-constrained settings when facilities have the right tools and supports. Even at the foundational levels of the health system, strong leadership and high management capacity are crucial signals of best performance. Further investigation of the practices that generate effective health facility leaders and replicating these conditions for excellence might be instrumental in strengthening primary care performance.

Positive deviance analysis, which compares the practices of best-performing organisations with low-performing organisations to identify strategies for success, can be used to understand variations in performance.^12^ Beyond identifying correlates of good quality, positive deviance analysis identifies factors that differentiate best from worst or low performance among facilities operating in similar contexts. Most positive deviance analyses focus on secondary care in high-income settings.^13^ Many are solely qualitative in nature, providing deep insight on a small number of facilities with little generalisability within and between countries.^14^ Additionally, there is more work on what drives success in high-performing facilities than on what factors underlie low performance.^14^ We modified the positive deviance approach to explore factors associated with best and worst performance in the provision of primary care services using nationally representative health system surveys from six LMICs. We aimed to develop a framework of potential performance factors and use the positive deviance approach to understand which of these factors explain gaps in performance. This approach is frequently applied in health systems in which average performance is of acceptable quality. Given prevailing low quality of care in the health systems included in this analysis, we adapted the positive deviance model to compare the best-performing facilities with health facilities performing at the average and below. Our findings could help to identify scalable practices that can improve primary care quality in resource-constrained health systems.

## Methods

### Study sample

This positive deviance analysis used data from Service Provision Assessments (SPAs), which are nationally representative surveys of health facilities conducted by the Demographic and Health Surveys Program. SPAs include an audit of facility resources, surveys on clinical practices, and direct observations of antenatal care, family planning care, and care for sick children. To identify client visits for observation, SPA surveyors used systematic random sampling to select a maximum of five clients for each provider of the specific service, with a maximum of 15 observations for each service in any given facility. Selected client visits were assessed in their entirety by trained observers. The SPA sampling strategy and methods have been described previously.^15^, ^16^

We used the most recent survey from each country that conducted an SPA in the past 10 years. We excluded SPA datasets that were older than 10 years, had no direct observations of care, only surveyed hospitals, or for which the data are publicly inaccessible. Our final sample included SPA data for the following countries: the Democratic Republic of the Congo (data collection Oct 16, 2017, to April 20, 2018), Haiti (Dec 16, 2017, to May 9, 2018), Malawi (June 11, 2013, to Feb 7, 2014), Nepal (April 20, 2015, to Nov 5, 2015), Senegal (April 15, 2019, to Feb 28, 2020), and Tanzania (Oct 20, 2014, to March 13, 2015). We included all health facilities surveyed in each SPA. We stratified surveyed facilities into hospitals and non-hospitals (which we refer to as clinics) on the basis of whether the facility conducts caesarean sections, a proxy measure for surgical capacity.

All research procedures were approved by the Harvard TH Chan School of Public Health Institutional Review Board.

### Outcomes

To assess the performance of facilities we used the Good Medical Practice Index (GMPI), which we previously developed to capture a minimum set of clinical activities required for making a diagnosis and proposing correct condition management (appendix pp 1–2).^7^ The index counts the completion of basic clinical activities covering history taking (eg, asking the patient's age), physical examination (eg, weighing the patient), and counselling (eg, warning of danger signs) that should be conducted for most patients presenting with a health problem. The index covers antenatal care (ten items), family planning care (eight items), and care for sick children (ten items) based on items asked in all SPAs matched with existing clinical guidelines. History-taking items might not apply to follow-up visits in antenatal care, so these items were excluded for relevant observations. Antenatal care was excluded for Senegal because direct observations of this service were not conducted in 2019. A facility's index score was calculated as the mean of the proportion of index items clinicians completed across patient encounters. The resulting facility-level score ranged from 0 to 100, with a higher score corresponding to greater performance of essential clinical actions.

To identify the best-performing and the worst-performing facilities of different levels, we separately pooled hospitals and clinics across countries and calculated deciles of the GMPI. We designated facilities in the top 10% of the index as the best performers and facilities below the median as the worst performers. A 10% threshold has been used in previous positive deviance analyses and aligns with the distribution of the outcome variable.^13^ Our primary outcome was a binary indicator for status as a best-performing facility versus a worst-performing facility across countries.

### Covariates

To identify factors that contribute to health facility performance, we created a conceptual framework based on previously identified foundations of high-quality health systems with four domains: population, governance, workforce, and tools. The platforms domain is unmeasured in SPA data and was excluded from this analysis (appendix pp 3–5).^6^ We mapped available SPA indicators to the framework, resulting in 14 measured potential factors influencing health facility performance and four contextual factors. Indicators were binary for facility-level factors and proportions for workforce factors and tools. The full definition of each performance factor is available in the appendix (p 5). In brief, management meetings were defined as having regular meetings, having a record of meetings, and taking actions in response; external supervision was defined as whether an external supervisor performed a set of 11 supervisory activities, such as checking facility registers and observing clinical care; basic amenities were measured as the average of seven items (electricity, water, any private room, toilet, communication, computer and internet, and ambulance); and service readiness was measured as the average of indices for each service area (care for sick children, antenatal care, and family planning care) with indicators covering basic equipment, diagnostics, and medication. Given the potential for overlap between related performance factors, we calculated the mean of the variables in each domain to create four facility-level summary scores. We included these four performance dimensions as the primary covariates of interest in our regression models. Because the platforms domain included functions beyond the facility locus of control, it is largely unmeasured in SPA data and was not included in our analysis.

### Statistical analysis

We compared characteristics of best-performing and worst-performing hospitals and clinics in descriptive analysis. To assess quality, we calculated the mean (SD) and median (IQR) of GMPI scores among the best and worst performers. We also calculated the levels of each performance dimension and its individual components in both samples, using *F*-tests and χ^2^ tests to assess the significance of differences between best and worst performers.

We constructed two multivariable logistic regression models to obtain adjusted odds ratios (ORs) and confidence intervals for the association between each performance dimension and status as best performer. The first model assessed associations between dimensions of performance and the likelihood of being a best-performing hospital compared with being a worst-performing hospital. The second model assessed the same associations among clinics. Our models included robust standard errors and controlled for facility contextual characteristics that are likely to influence performance and confound the relationship of interest, and country fixed effects to control for unobserved national factors such as health system strength. Nepal, which had the lowest average GMPI score among both hospitals and clinics, was used as a reference country.

To assess the sensitivity of the results to different specifications, we applied our main regression models to a sample of the top and bottom 10% of facilities pooled across countries as in a traditional symmetrical positive deviance analysis. We also tested a 5% and 15% threshold for best performance. Because the Democratic Republic of the Congo constituted a large portion of the hospital sample, we also ran our main models excluding this country. We did not weight estimates because this analysis did not aim to represent the health system of any particular country. All analyses were carried out using Stata version 16.1.

### Role of the funding source

The funder of the study had no role in study design and conduct, data collection, data management, data analysis, data interpretation, or writing of the report.

## Results

The SPA surveys included 4891 health facilities across the six countries with at least one direct observation of care and complete data on the predictor variables (appendix p 6). Of these, 1331 facilities were defined as hospitals and 3560 facilities were defined as clinics on the basis of surgical capacity. Of the hospitals, we identified 132 (10%) as best performing and 664 (50%) as worst performing. Of the clinics, we identified 355 (10%) as best performing and 1778 (50%) as worst performing.

**Figure 1:**
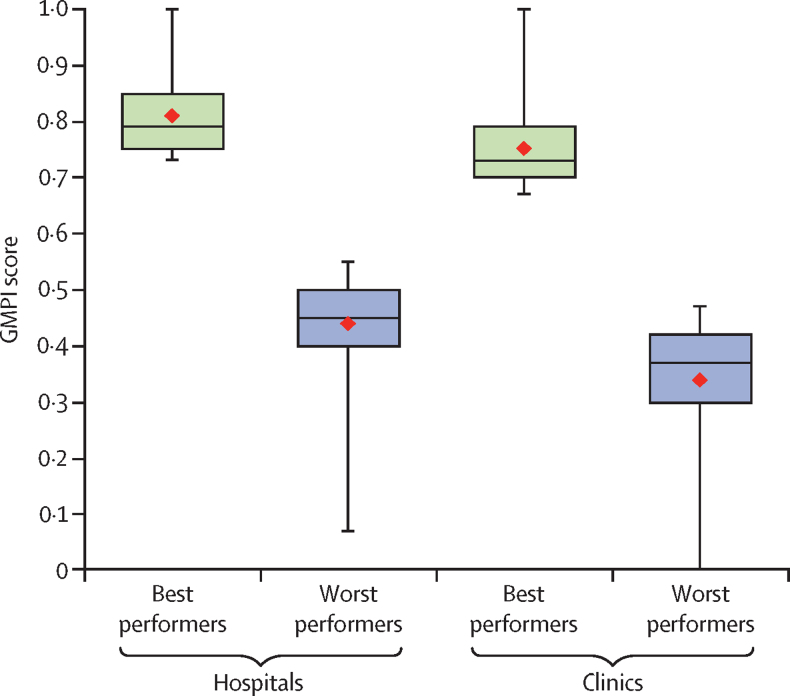
GMPI score among health facilities by performance status in six countries, 2013–19 GMPI score was calculated for 796 hospitals and 2133 clinics. Diamonds indicate mean performance. GMPI=Good Medical Practice Index.

**Figure 2:**
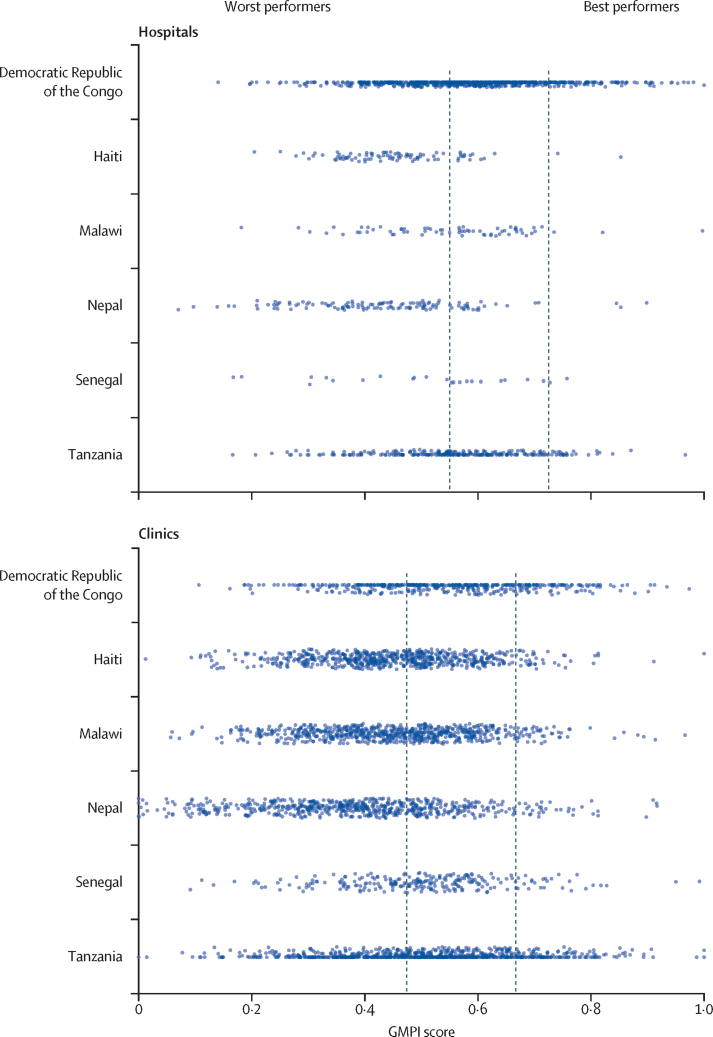
Distribution of GMPI scores for hospitals and clinics in six countries, 2013–19 GMPI score was calculated for 796 hospitals and 2133 clinics. Dashed lines indicate GMPI score at cutoffs for best and worst performance. GMPI=Good Medical Practice Index.

**Figure 3:**
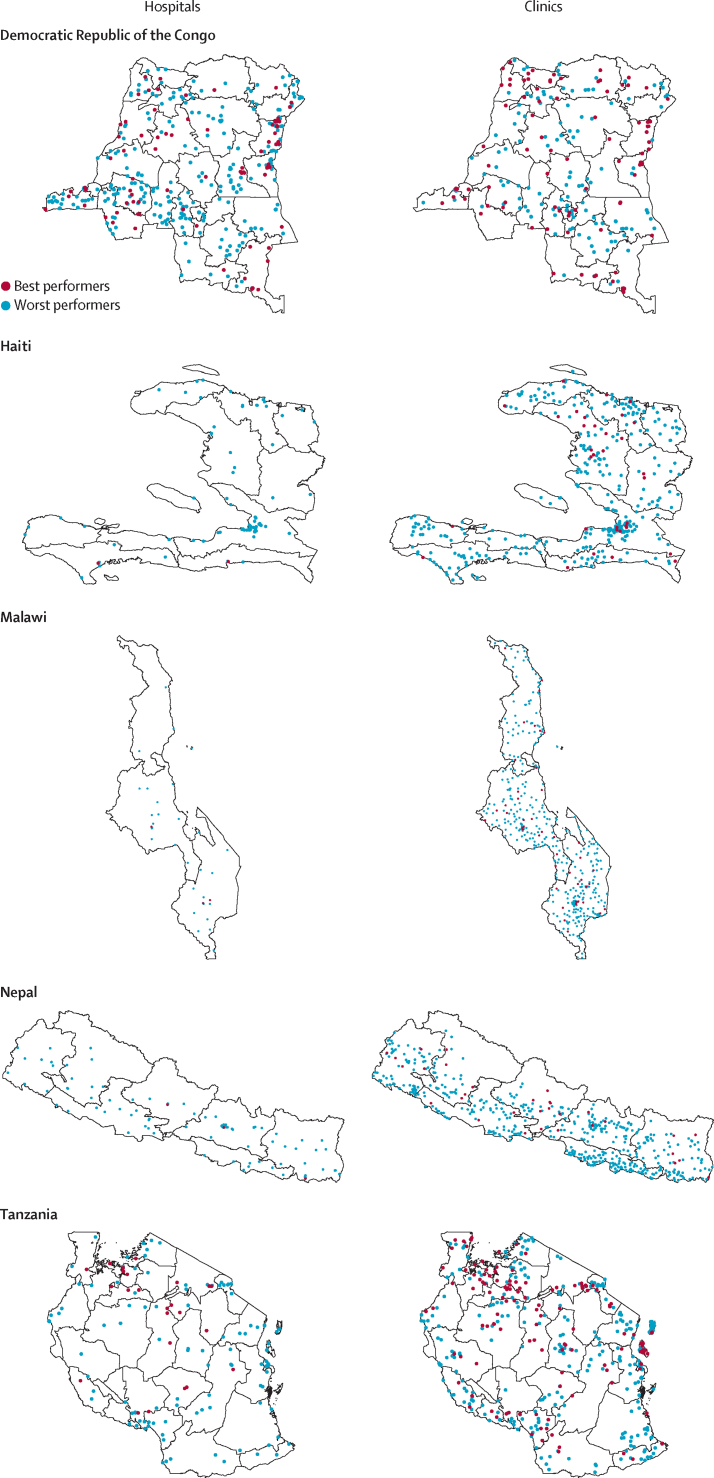
Geographic distribution of best-performing and worst-performing hospitals and clinics in five countries, 2013–18 GMPI score was calculated for 783 hospitals and 2016 clinics. Senegal was excluded from this analysis because geocodes were unavailable for the 2019 Service Provision Assessment.

Among the best-performing hospitals, 64 (48%) were public facilities and 42 (32%) were in urban areas (table 1). Among the best-performing clinics, 229 (65%) were public facilities and 110 (31%) were in urban areas. The mean GMPI score was 0·81 (SD 0·07) for the best-performing hospitals and 0·44 (0·09) for the worst-performing hospitals. Among clinics, mean GMPI scores were 0·75 (0·07) for the best performers and 0·34 (0·10) for the worst performers (figure 1). 99 (75%) best-performing hospitals and 320 (48%) worst-performing hospitals were in the Democratic Republic of the Congo, which comprised 57% of hospitals in the full sample (table 1; figure 2; appendix p 6). By contrast, best-performing and worst-performing clinics were more evenly distributed between countries. Facility locations were widely distributed throughout subnational regions in each country (figure 3).

**Table 1:** Characteristics of health facilities by performance status in six countries, 2013–19

		**Hospitals**		**Clinics**	
		Best performers (n=132)	Worst performers (n=664)	Best performers (n=355)	Worst performers (n=1778)
Mean client visits		12 (13)	26 (48)	11 (13)	13 (31)
Urban		42 (32%)	351 (53%)	110 (31%)	671 (38%)
Facility ownership					
	Government	64 (48%)	366 (55%)	229 (65%)	1221 (69%)
	Private not-for-profit	48 (36%)	190 (29%)	73 (21%)	273 (15%)
	Private for-profit	20 (15%)	108 (16%)	53 (15%)	284 (16%)
Service Provision Assessment country					
	Democratic Republic of the Congo	99 (75%)	320 (48%)	96 (27%)	142 (8%)
	Haiti	2 (2%)	75 (11%)	33 (9%)	373 (21%)
	Malawi	3 (2%)	31 (5%)	44 (12%)	402 (23%)
	Nepal	3 (2%)	103 (16%)	32 (9%)	456 (26%)
	Senegal	1 (1%)	12 (2%)	24 (7%)	93 (5%)
	Tanzania	24 (18%)	123 (19%)	126 (35%)	312 (18%)
Mean GMPI score		0·81 (0·07)	0·44 (0·09)	0·75 (0·07)	0·34 (0·10)

Data are mean (SD) for continuous measures and n (%) for categorical measures. GMPI score was calculated as a proportion of essential clinical actions (see appendix pp 1–2 for components). Client visits was defined as the number of client visits on the day of the survey. Totals might not add to 100% due to rounding. GMPI=Good Medical Practice Index.

**Figure 4:**
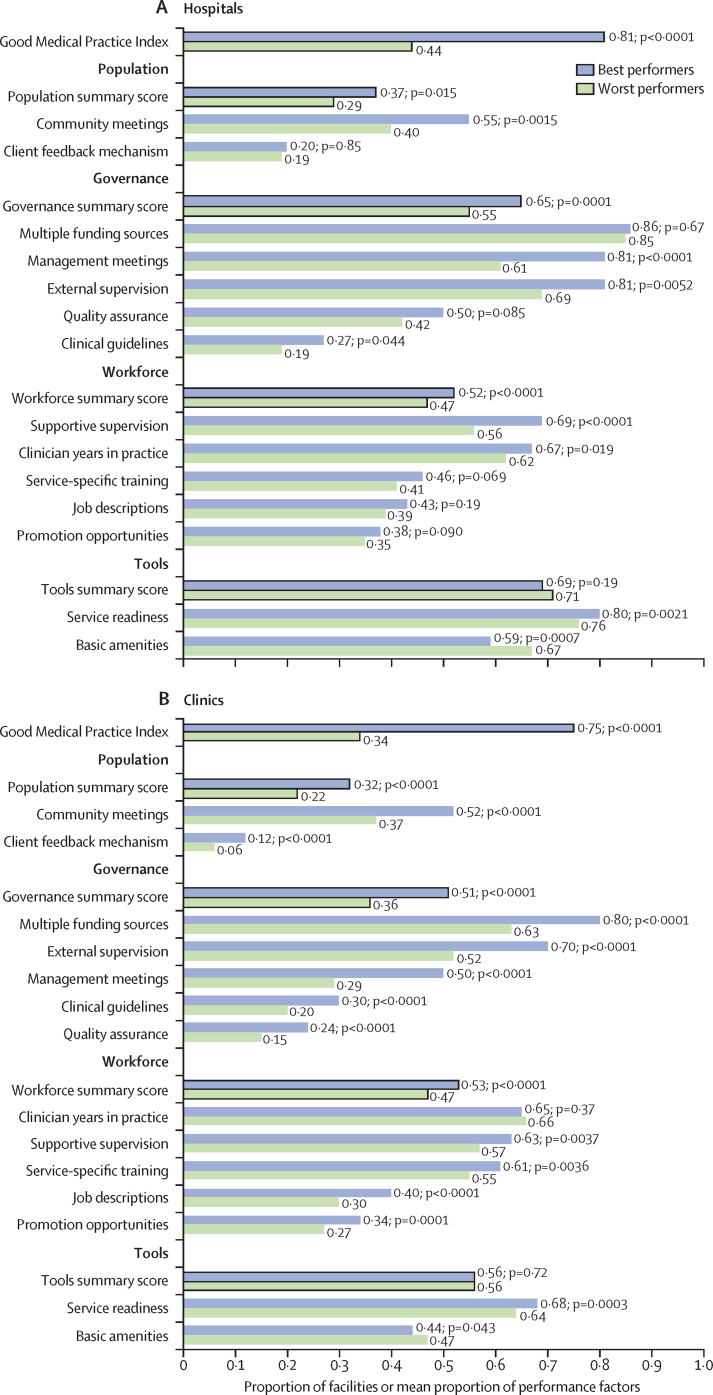
Performance factors among facilities in six countries, 2013–19 (A) Performance factors among hospitals (n=796). (B) Performance factors among clinics (n=2133). p values are for the comparison between best-performing and worst-performing facilities using *F*-tests for continuous variables and χ^2^ tests for categorical variables.

Best-performing hospitals significantly outperformed worst-performing hospitals on the population, governance, and workforce dimensions of performance (figure 4A). The largest difference was in governance: the best performers had a summary governance score of 0·65 (SD 0·24) compared with 0·55 (0·26) for the worst performers (p=0·0001). The best performers were more likely to have high-quality management meetings and follow-up (107 [81%] *vs* 408 [61%]; p<0·0001), external supervision (107 [81%] *vs* 458 [69%]; p=0·0052), and supportive supervision for clinicians (69% [SD 0·24] *vs* 56% [0·24]; p<0·0001).

Best-performing clinics outperformed worst-performing clinics on most factors, with significant differences for three of the four summary performance dimensions and 13 of 14 individual factors (figure 4B). As with the hospitals, the largest difference between the best-performing and worst-performing clinics was in the governance dimension, with a mean score of 0·51 (SD 0·25) among best performers and 0·36 (0·25) among worst performers (p<0·0001). Among individual factors, the best-performing clinics were more likely to have more sources of funding (283 [80%] *vs* 1123 [63%]; p<0·0001) and thorough external supervision (247 [70%] *vs* 930 [52%]; p<0·0001) than the worst-performing clinics. 40% (SD 0·30) of best performers had job descriptions for staff, whereas only 30% (0·28) of worst performers did (p<0·0001).

When testing associations between dimensions of facility performance and status as a best-performing versus worst-performing hospital, only the tools dimension was significantly associated with best performance (adjusted OR 1·37 [95% CI 1·01–1·85]; table 2). However, multiple contextual factors, including ownership type and number of client visits, were associated with being a best-performing hospital. Private for-profit hospitals had significantly greater odds of being best performers than government-run hospitals (table 2).

**Table 2:** Factors associated with best performance among health facilities in six countries, 2013–19

	**Hospitals (n=796)**		**Clinics (n=2133)**	
	Odds ratio (95% CI)	p value	Odds ratio (95% CI)	p value
**Performance dimensions**				
Population	1·09 (0·91–1·30)	0·37	1·16 (1·01–1·32)	0·030
Governance	1·22 (0·92–1·60)	0·17	1·22 (1·04–1·44)	0·015
Workforce	1·32 (0·98–1·79)	0·067	1·35 (1·18–1·54)	<0·0001
Tools	1·37 (1·01–1·85)	0·043	1·43 (1·21–1·70)	<0·0001
**Context**				
Urban	0·75 (0·45–1·25)	0·27	0·73 (0·52–1·04)	0·082
Private not-for-profit	1·08 (0·69–1·70)	0·74	1·84 (1·27–2·65)	0·0011
Private for-profit	1·90 (1·02–3·55)	0·042	1·67 (1·09–2·54)	0·017
Client education	1·00 (0·52–1·91)	0·99	1·04 (0·63–1·72)	0·87
Client visits	0·99 (0·98–1·00)	0·031	1·00 (0·99–1·00)	0·45
**Country (reference Nepal)**				
Democratic Republic of the Congo	7·40 (1·98–27·59)	0·0029	8·16 (4·68–14·22)	<0·0001
Haiti	0·73 (0·11–4·80)	0·74	0·91 (0·51–1·61)	0·74
Malawi	4·65 (0·82–26·43)	0·083	1·14 (0·66–1·97)	0·65
Senegal	1·51 (0·12–18·52)	0·75	2·09 (1·07–4·07)	0·031
Tanzania	4·36 (1·21–15·76)	0·025	5·54 (3·32–9·24)	<0·0001

Estimates were obtained using logistic regression with robust standard errors.

Among clinics, we found that the four summary performance dimensions were significantly associated with best performance (table 2). Both private for-profit and not-for-profit clinics were more likely than government clinics to be best performers. In both hospital and clinic models, facilities in the Democratic Republic of the Congo and Tanzania were more likely to be best performers.

Results from sensitivity analyses were largely consistent with those from the main models (appendix pp 7–10). Results from the model with a 5% threshold for best performance were similar to the model with a 10% threshold, although at a 5% cutoff, no hospitals in Senegal performed well enough to be included in the sample of best performers. In the hospital model with a 15% threshold, the workforce dimension was significant, while the tools dimensions was not, which is probably due to the decrease in variation between best and worst facilities at this less stringent performance standard.

## Discussion

We used routinely collected health system data from six LMICs to identify best-performing and worst-performing health facilities and facility-level factors that explain the performance gap. We found large differences in clinical quality between best-performing and worst-performing facilities. We also found an uneven distribution of best and worst performers between countries, reflecting highly variable health system quality at the national level. When considering factors previously posited as essential to performance, we found large differences in the structure and operations of facilities in similar contexts. In models adjusted for contextual characteristics, we found all four dimensions of performance remained significantly associated with best performance among clinics. However, among hospitals, only the tools dimension and contextual characteristics such as private ownership, client volumes, and country fixed effects were associated with the gap between best and worst performance.

These results reflect variation in basic functions that characterise excellent performance, especially at the level of the primary care clinic. For primary care facilities, local inputs matter: even when adjusting for contextual characteristics, best performance is driven or reflected by high-quality management, external and internal supervision, and other operational processes. However, among hospitals these inputs were not significantly associated with best performance. This suggests that common interventions to improve quality of care, such as in-service training or supportive supervision for clinicians, are unlikely to bridge the performance gap among hospitals in LMICs. For hospitals, which are typically better resourced and more likely to have established management and quality assurance procedures in place, it is likely that macro structures, such as local and national health system features, are more influential on performance. Thus, the level of intervention is crucial for identifying appropriate strategies to improve quality of care.

Importantly, performance dimensions identified in this analysis might reflect rather than generate best performance. Without longitudinal observation of facility performance, we are unable to determine causality. Many additional factors, such as leadership capacity, health system regulation, infrastructure, and subnational health policies, which are not measurable in SPAs, contribute to performance. However, this analysis identifies unifying factors that build a richer profile of strong primary care facilities and provides a starting point for identifying specific strategies for clinical excellence already in use locally that governments can bring to scale.

In our models, we found that governance was a key differentiator between hospitals and clinics in bivariable analyses and for clinics in fully adjusted models. Important factors included high-quality management meetings, strong external supervision, quality assurance mechanisms, having clinical guidelines, and obtaining multiple sources of funding. These factors reflect the important role of leaders who engage their staff, act on the basis of feedback, and make transparent decisions. These leaders also ensure availability of essential resources for top performance, including up-to-date clinical information and reliable sources of revenue.

Workforce factors, a by-product of good management, were also highly associated with best versus worst performance. Supportive supervision, a significant predictor of best performance in clinics in our models, can improve performance and serve as a mechanism for professional development, job satisfaction, and clinician motivation.^17^ As supervision is already ubiquitous, the primary challenge is to provide adequate support to managers to improve the quality of supervision and maximise effectiveness. This is particularly relevant for higher-level facilities for which common management strategies such as supportive supervision delivered in the standard manner did not explain the gap between best and worst performers.

Our results also suggest that clear role delineation and opportunities for training and promotion are significantly associated with best performance. Although these have been proposed as important interventions for strengthening health worker performance, micro-level strategies such as printed information, guidelines, and job aids are unlikely to improve clinical care beyond the existing relatively low standard of best performance. Similarly, in-service training programmes typically yield only moderate gains in care quality.^18^ Some evidence suggests these interventions might be more effective when delivered in combination with management strategies such as supportive supervision, although system-level reforms might still be required to meaningfully improve quality.^17^

We also found that clinics that had a client feedback mechanism and held and recorded a meeting with community members in the past 6 months were more likely to be best performers. This finding might reflect the important role of community accountability in primary care performance. Previous positive deviance analyses have found that managers and clinical staff in best-performing facilities reported feeling more accountable to community members than did staff in worst-performing facilities.^19^ This finding re-enforces existing evidence that community engagement and formal linkage with a health facility, such as through health facility committees, might have positive effects on primary care quality.^20^ The role of communities is likely to differ between public and private facilities, although our results suggest responsiveness to the community is important to performance regardless of ownership type.

The tools domain, composed of equipment, diagnostics, and medication for each service area, was significantly associated with best performance in both hospitals and clinics despite similar availability between best and worst performers in unadjusted analyses. This suggests that best-performing and worst-performing hospitals are differentiated by small but significant differences in supplies conditional on other factors. Although supplies are essential to care provision, they are no guarantee of high-quality care.^21^ It might be that facilities with strong management and leadership are better able to ensure adequate infrastructure in the facility, rather than infrastructure leading to strong medical practice. The role of leadership capacity in maintaining service readiness warrants further study.

These performance factors are united by the need for high-quality management, a frequently cited driver of best performance and an area worthy of investment by governments.^10^, ^22^, ^23^, ^24^ A study in Ghana, for example, found that better facility management was associated with trust in providers, ease of following a provider's advice, and overall quality rating.^23^ Management activities such as facility planning, target setting, performance tracking, and problem solving have been shown to be essential for differentiating best-performing and worst-performing facilities.^24^ Management accountability mechanisms, such as performance management systems, quality monitoring, and health information systems, are closely linked with clinician adherence to guidelines, which can promote high-quality clinical practice.^10^, ^22^, ^25^

Finally, we found private for-profit hospitals and clinics and private not-for-profit clinics were significantly more likely to be best performers than government owned facilities. Quality often differs between public and private facilities, though this varies by setting.^26^ However, for basic factors associated with excellent performance, private facilities are out-performing their government-run counterparts in these six countries. Nonetheless, our findings show that good performance can be located within different geographic settings and among private for-profit, not-for-profit, and public systems. The literature on quality variation would benefit from expanding beyond common measures of ownership and geographical location to understand what drives performance. Our results are also supportive of the growing shift in focus to the structural factors that influence good quality.^6^ Despite large clinical performance gaps, we found relatively small differences for most measured performance factors, suggesting that broader health system characteristics might be differentiating best from worst performance.

This study has several limitations. SPAs offer only a limited set of indicators that do not measure the full range of potential factors associated with facility performance.^11^ Given varying facility nomenclature, we used caesarean section as a proxy for hospitals (versus lower level clinics), although this might misclassify health facilities in areas where primary care facilities carry out surgeries due to low hospital access. The relationships between potential factors and best performance are associations and might reflect rather than determine facility performance. Unobserved factors, such as health system organisation or financing, might confound regression estimates. Further exploration of these associations is warranted.

Our study shows substantial performance gaps between facilities across countries despite frequently operating in similar contexts. This finding suggests that good medical practice is possible in resource-constrained settings when facilities have the right tools and supports. For lower-level facilities, these inputs include strong management, a supported workforce, supplies and equipment, and close linkages with the community. Local examples of these best practices are available and should be studied by leaders to replicate conditions for excellence more widely. Among hospitals, only adequate tools and private ownership were associated with better medical practice. Additional research is needed to identify specific pathways to higher quality for these facilities.

It is important to note that facility-level performance is heavily influenced by health system factors, such as governance and financing, that are determined at district, province, and national levels and might not be modifiable by health facilities.^6^ Identified facility-level factors, if effective, can only increase facility performance so much, especially among higher-level facilities that are less likely to benefit from microlevel quality improvement strategies; elevating quality in the country as a whole will require large-scale, upstream improvements to the health system.^6^ As countries progress towards universal health coverage, governments should look to best-performing health facilities to identify scalable best practices and new opportunities for quality improvement in primary care.

## Data sharing

The data used in this study are publicly available from the Demographic and Health Surveys Program at https://dhsprogram.com/data/available-datasets.cfm.

## Declaration of interests

We declare no competing interests.
