# Supplementary material for: Health service quality in 2929 facilities in six low-income and middle-income countries: a positive deviance analysis
Source: Lancet Glob Health. 2023 May 16;11(6):e862–70. doi: 10.1016/S2214-109X(23)00163-8 (PMC10205971; doi:10.1016/S2214-109X(23)00163-8)
Supplement: Supplementary appendix [file mmc1.pdf]

THE LANCET
Global Health

Supplementary appendix

This appendix formed part of the original submission and has been peer reviewed. We post it as supplied by the authors.

Supplement to: Lewis TP, McConnell M, Aryal A, et al. Health service quality in 2929 facilities in six low-income and middle-income countries: a positive deviance analysis. *Lancet Glob Health* 2023; **11:** e862–70.

Appendix table 1: Facility performance by Good Medical Practice Index clinical action item in six countries, 2013-2019

Clinical action item	Hospitals				Clinics			
	Best (n=132)		Worst (n=664)		Best (n=355)		Worst (n=1,788)	
	Mean	(SD)	Mean	(SD)	Mean	(SD)	Mean	(SD)
**Antenatal care**								
History-taking								
Asks client age	0.94	(0.18)	0.69	(0.40)	0.96	(0.17)	0.68	(0.43)
Asks number of past pregnancies	0.83	(0.30)	0.63	(0.39)	0.83	(0.31)	0.62	(0.43)
Asks date of last menstrual period	0.97	(0.12)	0.82	(0.32)	0.95	(0.18)	0.77	(0.37)
Asks about bleeding in pregnancy	0.64	(0.36)	0.19	(0.29)	0.59	(0.41)	0.12	(0.26)
Examination								
Checks for anemia	0.88	(0.22)	0.45	(0.39)	0.81	(0.32)	0.4	(0.42)
Measures fundal height	0.97	(0.12)	0.71	(0.38)	0.94	(0.20)	0.58	(0.45)
Measures blood pressure	0.98	(0.07)	0.71	(0.39)	0.93	(0.22)	0.67	(0.44)
Measures weight	0.96	(0.17)	0.76	(0.36)	0.92	(0.24)	0.67	(0.44)
Counseling								
Encourages questions	0.85	(0.29)	0.51	(0.42)	0.82	(0.33)	0.47	(0.45)
Counsels on 1+ danger signs	0.59	(0.42)	0.25	(0.31)	0.67	(0.41)	0.22	(0.34)
**Family planning**								
History-taking								
Asks client age	0.93	(0.20)	0.57	(0.40)	0.87	(0.30)	0.42	(0.42)
Asks desired timing of next child	0.56	(0.42)	0.11	(0.24)	0.33	(0.41)	0.05	(0.16)
Asks about STI symptoms	0.61	(0.41)	0.06	(0.19)	0.34	(0.43)	0.04	(0.15)
Asks date of last menstrual period	0.86	(0.33)	0.42	(0.38)	0.83	(0.30)	0.29	(0.37)
Examination								
Measures blood pressure	0.95	(0.13)	0.44	(0.44)	0.81	(0.37)	0.39	(0.44)
Measures weight	0.89	(0.26)	0.46	(0.43)	0.75	(0.40)	0.35	(0.43)
Counseling								
Asks about concerns w/ method	0.62	(0.46)	0.42	(0.38)	0.80	(0.33)	0.34	(0.41)
Counsels on 1+ issues on 1+ methods	0.94	(0.20)	0.75	(0.33)	0.93	(0.21)	0.66	(0.41)
**Sick child care**								
History-taking								
Asks about ability to drink	0.49	(0.40)	0.21	(0.28)	0.52	(0.41)	0.15	(0.26)
Asks about fever	0.98	(0.09)	0.80	(0.28)	0.96	(0.14)	0.76	(0.31)
Asks about sick feeding pattern	0.49	(0.40)	0.19	(0.28)	0.51	(0.41)	0.12	(0.24)
Asks about cough/trouble breathing OR vomiting	0.92	(0.20)	0.73	(0.30)	0.93	(0.20)	0.68	(0.35)
Examination								

Measures temperature	0.96	(0.13)	0.75	(0.35)	0.92	(0.18)	0.67	(0.39)
Assesses dehydration	0.55	(0.42)	0.20	(0.28)	0.49	(0.40)	0.10	(0.21)
Assesses respiration	0.61	(0.43)	0.18	(0.29)	0.41	(0.42)	0.13	(0.26)
Measures weight	0.76	(0.39)	0.56	(0.42)	0.57	(0.46)	0.38	(0.44)
Counseling								
States diagnosis	0.6	(0.41)	0.27	(0.33)	0.64	(0.41)	0.24	(0.34)
Counsels on 1+ danger signs	0.37	(0.43)	0.07	(0.17)	0.33	(0.39)	0.06	(0.17)

Notes: Three antenatal care history-taking items were assessed for first visits only: asks client age, asks number of past pregnancies, and asks date of last menstrual period.

Additional methods

To develop a conceptual framework of factors that may drive high-quality primary care performance, we reviewed organizational and management frameworks from health services research, business, and education. We synthesized indicators and domains common across these frameworks and mapped them to previously identified foundations of high quality health systems. The resulting conceptual model has five foundations, each composed of multiple domains that categorize specific performance factors measured at the facility level: 1) Population (the role of individuals, families, and communities), 2) governance (leadership, policies, financing, learning, and intersectoral action), 3) workforce (the role of facility managers and the health workforce), 4) platforms (care organization, connective systems), and 5) tools (hardware and software), and contextual factors (demographic, socioeconomic, and overall health system factors).

Appendix figure 1: Hypothesized facility characteristics associated with high-quality primary care

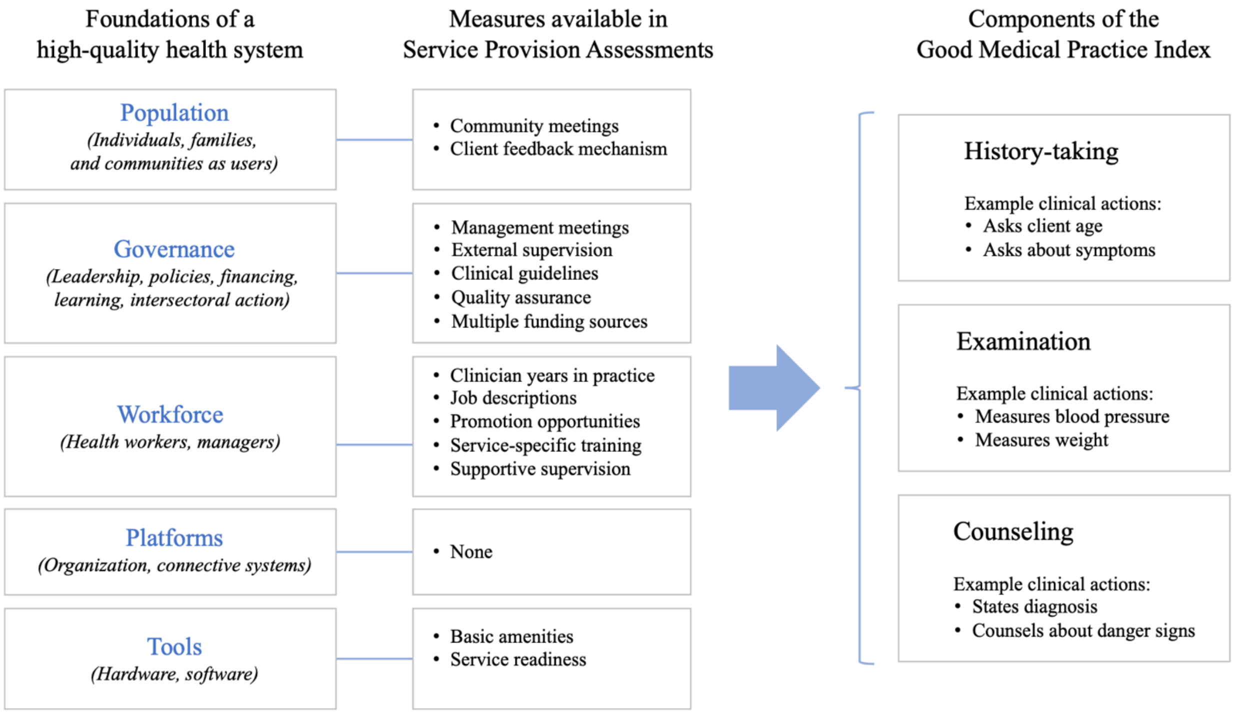

Appendix table 2: Definitions of performance characteristics from Service Provision Assessments

Variable	Definition
Population	
Community meetings	whether the facility held and recorded a meeting with community members in the past six months
Client feedback mechanism	whether the facility has system for obtaining, reviewing, and reporting clients' opinions
Governance	
Management meetings	whether the facility management meets at least every six months, made decisions based on the most recent meeting, and took action in follow up
External supervision	whether an external supervisor 1) conducted a supervisory visit in the last six months, 2) used a checklist to assess the quality of health services data, 3) discussed facility performance, 4) helped the facility make data-based performance decisions, 5) provided verbal or written feedback, 6) checked facility registers, 7) discussed performance problems, 8) discussed policy or administrative matters, 9) discussed technical protocols or service delivery issues, 10) held an official staff meeting, and 11) observed individuals providing clinical care
Clinical guidelines	whether the facility had service-specific guidelines for antenatal care, family planning, and sick child care
Quality assurance	whether the facility routinely carries out and records quality assurance activities
Multiple funding sources	whether the facility had multiple funding source available
Workforce	
Clinician years in practice	the proportion of clinicians with over five years' experience since graduation from training
Job descriptions	the proportion of clinicians with a written job description
Promotion opportunities	the proportion of clinicians who reported being aware of opportunities for promotion
Service-specific training	the proportion of clinicians with a service-specific training in the last six months
Supportive supervision	the proportion of clinicians who reported supervision that included discussion of problems encountered and receipt of supervisor feedback
Tools	
Basic amenities	the proportion of seven items available at a facility: electricity, water, any private room, toilet, communication, computer and internet, and ambulance
Service readiness	the proportion of essential basic equipment, diagnostics, and medication required in each service area
Contextual factors	
Urban	whether the facility is in an urban or rural location
Ownership	whether the facility managing authority is: 1) governmental, 2) non-governmental or not-for-profit, or 3) private for-profit
Client education	the proportion of clients with secondary school education or higher
Client visits	the number of client visits on the day of survey

Appendix table 3: Characteristics of health facilities included in Service Provision Assessments in six countries, 2013-2019

	**Hospitals**	**Clinics**
	N=1,331	N=3,560
**Client visits**	22 (38)	14 (26)
**Urban**	621 (47%)	1,285 (36%)
**Facility ownership**		
Government	712 (53%)	2,387 (67%)
Private not-for-profit	415 (31%)	644 (18%)
Private for-profit	204 (15%)	529 (15%)
**SPA country**		
DRC	755 (57%)	443 (12%)
Haiti	91 (7%)	684 (19%)
Malawi	67 (5%)	737 (21%)
Nepal	127 (10%)	650 (18%)
Senegal	24 (2%)	244 (7%)
Tanzania	267 (20%)	802 (23%)
**Technical quality:**		
Good medical practice index	0.55 (0.14)	0.47 (0.16)

Notes: Data are presented as mean (SD) for continuous measures, and n (%) for categorical measures. Good Medical Practice Index (GMPI) score is calculated as a proportion of essential clinical actions. See appendix for components. Hospitals were defined as facilities that perform Caesarean sections. Client visits was defined as the number of client visits on the day of the survey. Totals may not add to 100% due to rounding.

Appendix table 4: Factors associated with best performance among the best 10% and worst 10% of health facilities in six countries, 2013-2019

	**Hospitals**		**Clinics**	
Variables	Odds ratio	95% CI	Odds ratio	95% CI
**Performance dimensions**				
Population	1.02	0.73 - 1.42	1.15	0.93 - 1.43
Governance	1.40	0.89 - 2.21	1.51***	1.19 - 1.91
Workforce	1.56	0.97 - 2.53	1.24*	1.03 - 1.50
Tools	1.97**	1.18 - 3.28	1.64***	1.25 - 2.15
**Context**				
Urban	0.53	0.26 - 1.10	0.86	0.52 - 1.40
Private not-for-profit	0.96	0.47 - 1.96	2.14**	1.21 - 3.80
Private for-profit	1.67	0.59 - 4.73	2.55**	1.34 - 4.83
Client education	1.40	0.52 - 3.80	1.21	0.65 - 2.26
Client visits	0.99*	0.98 - 1.00	1.01*	1.00 - 1.03
**Country (ref: Nepal)**				
DRC	24.57***	4.05 - 149.05	20.07***	9.46 - 42.59
Haiti	0.85	0.09 - 8.35	1.88	0.91 - 3.90
Malawi	6.15	0.98 - 38.52	1.24	0.61 - 2.54
Senegal	0.88	0.05 - 17.17	3.28*	1.29 - 8.37
Tanzania	8.35*	1.63 - 42.86	8.24***	4.30 - 15.82
Observations	263		710	

*p<0.05 **p<0.01 ***p<0.001

Notes: Estimates were obtained using logistic regression with robust standard errors. Hospitals were defined as facilities that perform Caesarean sections. Best and worst performers were the top 10% and bottom 10% of facilities across countries based on Good Medical Practice Index (GMPI) score, a proportion of essential clinical actions. See appendix for components. Performance dimensions are summary indices constructed by averaging relevant items in each category at the facility-level. Population includes community meetings and client feedback mechanisms. Governance includes multiple funding sources, external supervision, management meetings, clinical guidelines, and quality assurance. Workforce includes clinician years in practice, supportive supervision, service-specific training, job descriptions, and promotion opportunities. Tools includes service readiness and basic amenities. Management meetings was defined as having regular meetings, having a record of meetings, making decisions during meetings, and taking actions in response. External supervision was defined as whether an external supervisor performed a set of 11 supervisory activities, such as checking facility registers and observing clinical care. See appendix for a full list of activities. Basic amenities were measured as the average of seven items: electricity, water, any private room, toilet, communication, computer and internet, and ambulance. Service readiness was measured as the average of indices for each service area (sick child care, antenatal care, family planning care) with indicators covering basic equipment, diagnostics, and medication. Client education was defined as the proportion of clients with secondary education or higher. Client visits was defined as the number of client visits on the day of the survey.

Appendix table 5: Factors associated with best performance among the best 5% and worst 50% of health facilities in five countries, 2013-2018

	**Hospitals**		**Clinics**	
Variables	Odds ratio	95% CI	Odds ratio	95% CI
**Performance dimensions**				
Population	1.19	0.93 - 1.54	1.15	0.96 - 1.36
Governance	1.08	0.75 - 1.54	1.24	1.00 - 1.55
Workforce	1.30	0.87 - 1.96	1.31**	1.11 - 1.55
Tools	1.59*	1.05 - 2.41	1.55***	1.23 - 1.94
**Context**				
Urban	1.07	0.52 - 2.21	0.91	0.57 - 1.46
Private not-for-profit	0.80	0.42 - 1.53	1.87**	1.18 - 2.97
Private for-profit	1.66	0.84 - 3.28	1.19	0.68 - 2.10
Client education	1.78	0.73 - 4.36	0.66	0.33 - 1.35
Client visits	0.95**	0.93 - 0.98	0.99	0.97 - 1.00
**Country (ref: Nepal)**				
DRC	5.89*	1.46 - 23.68	11.51***	5.39 - 24.58
Haiti	0.52	0.05 - 5.22	0.79	0.34 - 1.84
Malawi	7.37*	1.24 - 43.75	1.02	0.46 - 2.27
Tanzania	1.98	0.45 - 8.72	6.68***	3.34 - 13.36
Observations	718		1,956	

*p<0.05 **p<0.01 ***p<0.001

Notes: Estimates were obtained using logistic regression with robust standard errors. Hospitals were defined as facilities that perform Caesarean sections. Best and worst performers were the top 5% and bottom 50% of facilities across countries based on Good Medical Practice Index (GMPI) score, a proportion of essential clinical actions. See appendix for components. Performance dimensions are summary indices constructed by averaging relevant items in each category at the facility-level. Population includes community meetings and client feedback mechanisms. Governance includes multiple funding sources, external supervision, management meetings, clinical guidelines, and quality assurance. Workforce includes clinician years in practice, supportive supervision, service-specific training, job descriptions, and promotion opportunities. Tools includes service readiness and basic amenities. Management meetings was defined as having regular meetings, having a record of meetings, making decisions during meetings, and taking actions in response. External supervision was defined as whether an external supervisor performed a set of 11 supervisory activities, such as checking facility registers and observing clinical care. See appendix for a full list of activities. Basic amenities were measured as the average of seven items: electricity, water, any private room, toilet, communication, computer and internet, and ambulance. Service readiness was measured as the average of indices for each service area (sick child care, antenatal care, family planning care) with indicators covering basic equipment, diagnostics, and medication. Client education was defined as the proportion of clients with secondary education or higher. Client visits was defined as the number of client visits on the day of the survey. Senegal was excluded due to small sample.

Appendix table 6: Factors associated with best performance among the best 15% and worst 50% of health facilities in six countries, 2013-2019

	**Hospitals**		**Clinics**	
Variables	Odds ratio	95% CI	Odds ratio	95% CI
**Performance dimensions**				
Population	1.15	0.98 - 1.35	1.17**	1.04 - 1.31
Governance	1.08	0.85 - 1.36	1.24**	1.08 - 1.42
Workforce	1.50**	1.15 - 1.95	1.26***	1.12 - 1.42
Tools	1.29	0.99 - 1.67	1.45***	1.25 - 1.69
**Context**				
Urban	0.84	0.55 - 1.31	0.66**	0.49 - 0.90
Private not-for-profit	1.04	0.71 - 1.53	1.72***	1.25 - 2.36
Private for-profit	1.65	0.94 - 2.89	1.58*	1.10 - 2.26
Client education	1.09	0.63 - 1.91	1.15	0.75 - 1.74
Client visits	0.99*	0.98 - 1.00	1.00	1.00 - 1.00
**Country (ref: Nepal)**				
DRC	6.47***	2.23 - 18.80	8.07***	5.01 - 12.98
Haiti	0.46	0.08 - 2.55	1.07	0.66 - 1.72
Malawi	4.94*	1.25 - 19.58	1.23	0.77 - 1.96
Senegal	2.79	0.54 - 14.39	2.27**	1.24 - 4.14
Tanzania	4.75**	1.71 - 13.16	5.78***	3.73 - 8.96
Observations	863		2,301	

*p<0.05 **p<0.01 ***p<0.001

Notes: Estimates were obtained using logistic regression with robust standard errors. Hospitals were defined as facilities that perform Caesarean sections. Best and worst performers were the top 15% and bottom 50% of facilities across countries based on Good Medical Practice Index (GMPI) score, a proportion of essential clinical actions. See appendix for components. Performance dimensions are summary indices constructed by averaging relevant items in each category at the facility-level. Population includes community meetings and client feedback mechanisms. Governance includes multiple funding sources, external supervision, management meetings, clinical guidelines, and quality assurance. Workforce includes clinician years in practice, supportive supervision, service-specific training, job descriptions, and promotion opportunities. Tools includes service readiness and basic amenities. Management meetings was defined as having regular meetings, having a record of meetings, making decisions during meetings, and taking actions in response. External supervision was defined as whether an external supervisor performed a set of 11 supervisory activities, such as checking facility registers and observing clinical care. See appendix for a full list of activities. Basic amenities were measured as the average of seven items: electricity, water, any private room, toilet, communication, computer and internet, and ambulance. Service readiness was measured as the average of indices for each service area (sick child care, antenatal care, family planning care) with indicators covering basic equipment, diagnostics, and medication. Client education was defined as the proportion of clients with secondary education or higher. Client visits was defined as the number of client visits on the day of the survey.

Appendix table 7: Factors associated with best performance among health facilities in five countries (DRC excluded), 2013-2019

	Hospitals		Clinics	
Variables	Odds ratio	95% CI	Odds ratio	95% CI
**Performance dimensions**				
Population	1.00	0.70 - 1.43	1.09	0.93 - 1.27
Governance	0.90	0.45 - 1.78	1.31**	1.09 - 1.57
Workforce	0.97	0.46 - 2.03	1.35***	1.16 - 1.56
Tools	1.25	0.58 - 2.71	1.19	0.98 - 1.45
**Context**				
Urban	0.58	0.23 - 1.45	0.75	0.51 - 1.10
Private not-for-profit	1.13	0.39 - 3.26	2.12***	1.41 - 3.19
Private for-profit	1.66	0.57 - 4.86	1.57	0.97 - 2.56
Client education	0.68	0.14 - 3.34	1.33	0.73 - 2.42
Client visits	0.99	0.97 - 1.00	0.99	0.99 - 1.00
**Country (ref: Nepal)**				
Haiti	0.76	0.10 - 5.97	0.80	0.45 - 1.44
Malawi	4.82	0.73 - 31.59	1.13	0.64 - 2.01
Senegal	2.53	0.18 - 35.96	2.13*	1.07 - 4.27
Tanzania	5.56*	1.28 - 24.08	4.94***	2.91 - 8.38
Observations	377		1,895	

*p<0.05 **p<0.01 ***p<0.001

Notes: Estimates were obtained using logistic regression with robust standard errors. Hospitals were defined as facilities that perform Caesarean sections. Best and worst performers were the top 10% and bottom 50% of facilities across countries based on Good Medical Practice Index (GMPI) score, a proportion of essential clinical actions. See appendix for components. Performance dimensions are summary indices constructed by averaging relevant items in each category at the facility-level. Population includes community meetings and client feedback mechanisms. Governance includes multiple funding sources, external supervision, management meetings, clinical guidelines, and quality assurance. Workforce includes clinician years in practice, supportive supervision, service-specific training, job descriptions, and promotion opportunities. Tools includes service readiness and basic amenities. Management meetings was defined as having regular meetings, having a record of meetings, making decisions during meetings, and taking actions in response. External supervision was defined as whether an external supervisor performed a set of 11 supervisory activities, such as checking facility registers and observing clinical care. See appendix for a full list of activities. Basic amenities were measured as the average of seven items: electricity, water, any private room, toilet, communication, computer and internet, and ambulance. Service readiness was measured as the average of indices for each service area (sick child care, antenatal care, family planning care) with indicators covering basic equipment, diagnostics, and medication. Client education was defined as the proportion of clients with secondary education or higher. Client visits was defined as the number of client visits on the day of the survey.
